# Bone Plates Runout Prediction Through Tensile Strength and Geometric Properties for Regulatory Mechanical Testing

**DOI:** 10.1007/s10439-023-03363-2

**Published:** 2023-09-19

**Authors:** Federico Andrea Bologna, Alberto Luigi Audenino, Mara Terzini

**Affiliations:** 1 Department of Mechanical and Aerospace Engineering, Politecnico di Torino, Corso Duca degli Abruzzi, 24, 10129 Turin, Italy; 2 PolitoBIOMed Lab, Politecnico di Torino, Corso Duca degli Abruzzi, 24, 10129 Turin, Italy

**Keywords:** Osteosynthesis devices, ASTM F382, Four-point bending test, Fatigue analysis, Maximum bending moment

## Abstract

Mechanical tests on bone plates are mandatory for regulatory purposes and, typically, the ASTM F382 standard is used, which involves a four-point bending test setup to evaluate the cyclic bending fatigue performance of the bone plate. These test campaigns require a considerable financial outlay and long execution times; therefore, an accurate prediction of experimental outcomes can reduce test runtime with beneficial cost cuts for manufacturers. Hence, an analytical framework is here proposed for the direct estimation of the maximum bending moment of a bone plate under fatigue loading, to guide the identification of the runout load for regulatory testing. Eleven bone plates awaiting certification were subjected to a comprehensive testing campaign following ASTM F382 protocols to evaluate their static and fatigue bending properties. An analytical prediction of the maximum bending moment was subsequently implemented based on ultimate strength and plate geometry. The experimental loads obtained from fatigue testing were then used to verify the prediction accuracy of the analytical approach. Results showed promising predictive ability, with *R*^2^ coefficients above 0.95 in the runout condition, with potential impact in reducing the experimental tests needed for the CE marking of bone plates.

## Introduction

Nowadays the development of new plates for osteosynthesis, especially in long-bone surgery, has undergone a broad improvement. Their design has changed over the years, from conventional plates with straight linear geometries to implants with irregular shapes that follow the anatomical profile of the healing bone [5]. Indeed, the complexity of clinical procedures and biomechanical characteristics has given rise to a great variety of bone plate concepts (e.g., locking compression plates, dynamic compression plates, variable angle-locking compression plates) intended for specific anatomical regions [11].

Although modern plates must meet safety and performance requirements depending on the local biomechanics [8], there is an increasing number of case reports documenting the complications associated with this surgical approach [22]. Examples of these complications include screw loosening, plate exposure, and screw or plate fracture [13]. Research has shown that metallic fixation devices are often subjected to high stress, which can potentially cause either catastrophic overloading or cyclic fatigue [3]. For these purposes, different studies have focused on reproducing the post-surgical conditions that caused the implant to fail [23].

Strategies based on Finite Element (FE) analyses are usually proposed to study the effect of a hole [4, 16, 27] or a notch [26] in the stress distribution of a bone plate. Moreover, FE analyses are often used for the calculation of the stress distribution in a static condition, to be inserted into commercial software for the evaluation of fatigue life through analytical methods [1, 7]. Another application of FE analysis which employed conventional S–N curves (stress amplitude *vs.* number of cycles to failure) has been used to investigate the fatigue properties of hybrid reconstruction plates for segmental defects treatment of mandible [17, 28].

On the other hand, to the best of the authors' knowledge, no study has been conducted to support the experimental testing necessary for the certification of bone plates. As a matter of fact, manufacturers and research groups are used to evaluate bone plates before commercialization through test methods published as ASTM or ISO standards [21]. ASTM F382 [2] is recognized in full by Food and Drug Administration (FDA) and Medical Device Regulation (MDR), and it is the most widely used test standard for metallic bone plates. This standard presents a four-point bending test setup that allows the evaluation of the bending stiffness, the bending structural stiffness, the bending strength, and the cyclic bending fatigue performance of the bone plate. Quasi-static tests are useful to determine the loading range of fatigue testing and, finally, a logarithmic graph of the applied bending moment as a function of the number of cycles to failure should be created (M–N diagram).

Fatigue testing has a substantial impact on the total runtime of the experimental campaign. The standard suggests for fatigue tests a load frequency of 5 Hz and one million cycles to reach the runout condition. An overall test time per plate has been estimated at around 180 hours without interruption in the best-case scenario [24]. Indeed, the realization of these test campaigns requires a considerable financial outlay on the part of manufacturers, with very long execution times from the market point of view.

Moreover, the main goal of ASTM standards is to establish a reference test method that can be performed in any accredited laboratory, thus supplying objective results for direct comparison with predicate devices. ASTM F382 provides a test methodology and defines the setup condition, but no indication is provided on the fatigue strength that the fixation device should guarantee in the runout condition.

The first support comes from a recently issued guideline provided by FDA [8] about the performance criteria accepted for each intended anatomical location when ASTM F382 is applied. Nevertheless, this guideline only refers to static tests and does not contain any indication of plate fatigue limits. In this scenario, it would be useful to define a "harmonised testing approach", as highlighted in Schorler et al. [21]: a systematic procedure based on standardized criteria for the development of a test procedure. Because of the wide variety of implants and their features, the test procedures shall be developed for each application and must be compliant with the regulatory requirements of ISO 14602, 7.2 [9].

To address these issues, this work suggests an analytical framework aimed at directly estimating the maximum bending moment of a bone plate, to speed up the experimental tests campaign for regulatory purposes.

## Materials and Methods

### Standard Mechanical Testing

Eleven different bone plates were assessed in the present study. Table 1 lists the material, the static ultimate tensile strength (*σ*_UTS_), and the anatomical site for which each plate was designed. A wide variety of plates with different sections were selected in order to test the analytical procedure over the entire range specified by the FDA guidelines [8], from plates designed for distal extremities to those intended for femoral regions. The first three plates referred to the distal extremities, *Plate04* is a clavicular bone plate, and *Plate05* to *Plate11* are designed for the lower extremities. Indeed, according to the guidance “Orthopedic Fracture Fixation Plates—Performance Criteria for Safety and Performance Based Pathway” [8], bone plates for the hand and foot necessitate the least performance for static testing, while clavicle plates necessitate performance comparable to the distal tibia. The highest performance is required for the femur and proximal tibia.

**Table 1:** Bone plates involved in the study and their main characteristics

Plate	Material	*σ*_UTS_ (MPa)	Anatomical location
01	Pure Titanium	668	Wrist
02	Stainless steel	900	Malleolus
03	Ti_6_Al_4_V	966	Malleolus
04	Stainless steel	986	Clavicle
05	Stainless steel	910	Distal tibia
06	Stainless steel	960	Distal tibia
07	Ti6Al4V	1012	Distal tibia
08	Ti6Al4V	942	Diaphyseal tibia
09	Stainless steel	986	Distal femur
10	Ti6Al4V	968	Distal femur
11	Pure Titanium	991	Diaphyseal femur

In order to facilitate the straightforward implementation of the procedure, the value of *σ*_UTS_ reported in the raw material certification of each plate, directly provided by the manufacturers, has been utilized.

The methodology described in the ASTM F382 standard was applied for a complete experimental test campaign. Firstly, quasi-static four-point bending tests were performed in displacement control to determine suitable loading levels for the fatigue testing. According to A1.6.2.1, rigid extension segments were adopted for plates that did not directly fit into the test configuration, to obtain a sufficiently long section of symmetry.

**Fig. 1:**
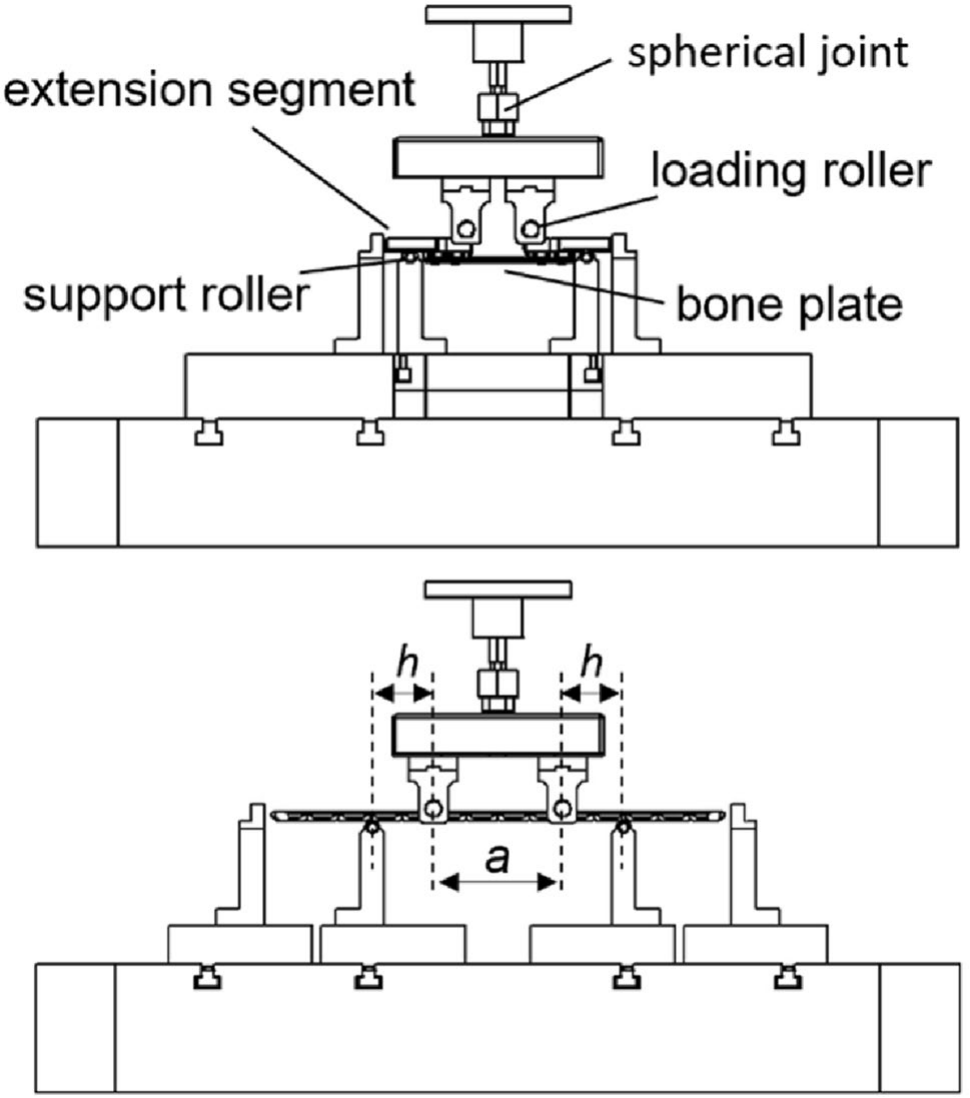
Main components of testing setup and characterizing in case rigid extension segments are either necessary (Top), or not required (Bottom)

The testing setup (Fig. 1) was composed of:

- two loading rollers with distance *a* (center span), at least two screw holes must be located between them;
- two support rollers, placed at an equal distance from the loading roller (*h*, loading span); and
- two extension segments (material AISI 630, heat treatment W1.4542 – H900), if required.

The loading rollers were rigidly connected to a spherical joint, while the support rollers were positioned on holders designed to be used with the testing machine base (Instron E3000, Norwood, MA, USA). Four plates of each model were tested until failure at 5 mm/min to assess the proof load, obtained from the intersection point of the load-displacement curve and the 0.2% offset displacement line.

The same setup was used in load control for fatigue tests to determine the runout load and the related maximum bending moment of every bone plate. The runout load (*L*) was determined starting from 90% of the proof load and gradually reducing it in discrete steps until reaching the load that led to one million cycles. Bending moments (*M*) were computed from the experimental runout load following Eq. 1:

1 $$M=L \frac{h}{2}$$

The runout condition was repeated on three different samples. As recommended by the ASTM standard, fatigue tests were performed at 5 Hz in load control, adopting an *R-ratio* of 0.1 (i.e., the minimum of the sinusoidal waveform was computed as 10% of the maximum load).

### Extraction of the Geometric Characteristics of the Plate

A customized MATLAB R2020b script (MathWorks, Natick, MA, USA) was employed to identify the critical section of each plate. The section with the least moment of inertia in the bending direction was selected as the critical section [25].

**Fig. 2:**
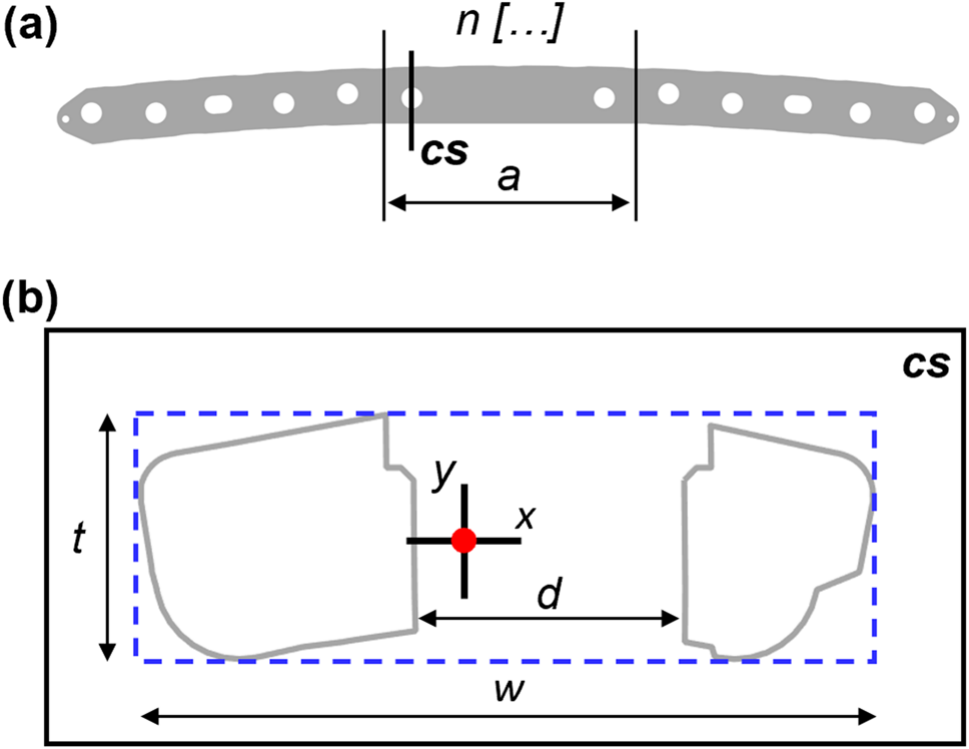
**a** Slicing technique applied to Plate09, where “*a*” is the center span required by the ASTM standard, “*n*” is the number of section planes, and “cs” is the identified critical section; **b** plate critical section main dimensions, red circle indicates the centroid of the section and blue dashed lines show the 2d-bounding box

First, the CAD files of the plates (*step* format) provided by the manufacturers were converted into point clouds (*stl* file) using SolidWorks 2020 (Dassault Systèmes, Vélizy-Villacoublay, France). The point clouds were oriented to align their longitudinal axis with the z-axis of the global system. Subsequently, the *geom3d* computing library [12] was exploited to generate the bounding box of the point set that constitutes the bone plate with the *boundingBox3d* function. The experimental bending test region (*a*) was divided with equidistant cutting plans (*n* = 100) along the longitudinal length of the box. To approximate the plate curvature, the centroid of the polyline resulting from the intersection between the plate and planes has been calculated for each section. Plate curvature was thus approximated by vectors connecting consecutive centroids. Following this latter, a new plate slicing was then performed with the same number of planes. Figure 2a shows the slicing technique applied for *Plate09*. At the location of a hole, the resulting section will consist of two distinct polylines (Fig. 2b). To determine the area and the centroid coordinates of each polyline, the function *PolygonMoments* [14, 19] was exploited. Centroid coordinates of the entire section were determined by applying Varignon's theorem, and then the Huygens-Steiner theorem was employed to ultimately determine the centroidal moment of inertia relative to the bending axis (${I}_{xx}$).

For each plate, the section with the smallest moment of inertia was automatically selected, as higher stresses are expected due to the reduced bending strength. In this section, the plate’s width (*w*) and thickness (*t*), as well as the hole’s minimum diameter (*d*) were automatically extrapolated (Fig. 2b). The function *boundingBox2d* from the *geom3d* library [12] was implemented to generate a rectangular box from the points on the section plane. The main dimensions of the box have been assumed as the thickness and width of the plate section. The inner diameter was evaluated as the minimum distance between the two polylines.

### Analytical Fatigue Limit Determination

The analytical evaluation of fatigue behaviour was carried out considering simplified straight plates, derived from the actual geometries by using the characteristics of their critical sections. Plate curvatures were neglected, while the presence of a screw hole was considered by applying the nominal tension Peterson’s stress concentration factors (${K}_{t}$) for transverse bending of a finite-width plate with a circular hole [18]. Indeed, in static loading, the stress distribution is amplified by the notch effect. In fatigue loading, empirical evidence indicates that the stress concentration factor diminishes at the fatigue limit [20]. In general, a fatigue-notch factor (${K}_{f}$) is often determined by modifying the value of ${K}_{t}$ (Eq. 2) according to a coefficient called notch-sensitivity index (*q*):

2 $${K}_{f}=1+q\left({K}_{t}-1\right)$$

The notch-sensitivity index depends on both the mechanical and metallurgical characteristics of the material and the absolute dimensions of the notch. Due to the large dispersion of available data, different equations have been proposed to obtain more precise values of *q*; the Neuber equation (Eq. 3) can be applied to steel plates:

3 $$q=\frac{1}{1+\sqrt{\rho }/\sqrt{r}}$$

where *ρ* is the average grain size and *r* is the notch radius.

While the Neuber equation is well-founded for steels, there is no clear relationship for titanium alloys. In these cases, it is mandatory to carry out fatigue tests on notched and unnotched specimens to determine ${K}_{f}$ [15]. Because materials with a uniform fine-grained matrix are very sensitive to notches [10], titanium plates ${K}_{f}$ was considered equal to ${K}_{t}$ (*q* = 1), from a conservative perspective.

Starting from the static ultimate tensile strength (${\upsigma }_{\mathrm{UTS}}$) specified in the raw material certification of the plate, ${K}_{f}$ was used to estimate the fatigue limit (${\upsigma }_{D-1}$) with no average stress (${\sigma }_{m}$ = 0, corresponding to *R-ratio* = − 1) of each bone plate from Eq. 4 [20]:

4 $${\upsigma }_{D-1}{=\upsigma }_{D-1}^{*}\frac{\prod {C}_{\mathrm{i}}}{{K}_{f}}$$

where ${\upsigma }_{D-1}^{*}$ is the fatigue limit of the specimen, while *C*_*i*_*s* are factors affecting the component fatigue life, such as size (*C*_S_), surface finish (*C*_F_), or loading mode (*C*_L_). In this study, a unit value has been attributed to $\prod {C}_{\mathrm{i}}$: no correction due to surface finish was applied (all surface roughnesses were less than 0.8 μm) and a reliability of 50% was considered. A fatigue limit equal to 0.5 ${\upsigma }_{\mathrm{UTS}}$ (Junival and Marshek, 2012) was applied for all bone plates.

Goodman and Gerber failure criteria [6] were implemented to calculate the mean stress effect due to ASTM F382 testing condition (*R*-ratio = 0.1). The conventional Goodman relationship (Eq. 5) includes the ultimate tensile strength (${\upsigma }_{\mathrm{UTS}}$) and was implemented in this study to define the lower limit of fatigue prediction.

5 $$\frac{{\upsigma }_{m}}{{\upsigma }_{\mathrm{UTS}}}+\frac{{\upsigma }_{a}}{{\upsigma }_{D-1}}=1$$

A usually non-conservative approach for tensile mean stresses [6] is the Gerber parabola (Eq. 6): this criterion was applied to define the upper limit of the fatigue prediction.

6 $${\left(\frac{{\upsigma }_{m}}{{\upsigma }_{\mathrm{UTS}}}\right)}^{2}+\frac{{\upsigma }_{a}}{{\upsigma }_{D-1}}=1$$

**Fig. 3:**
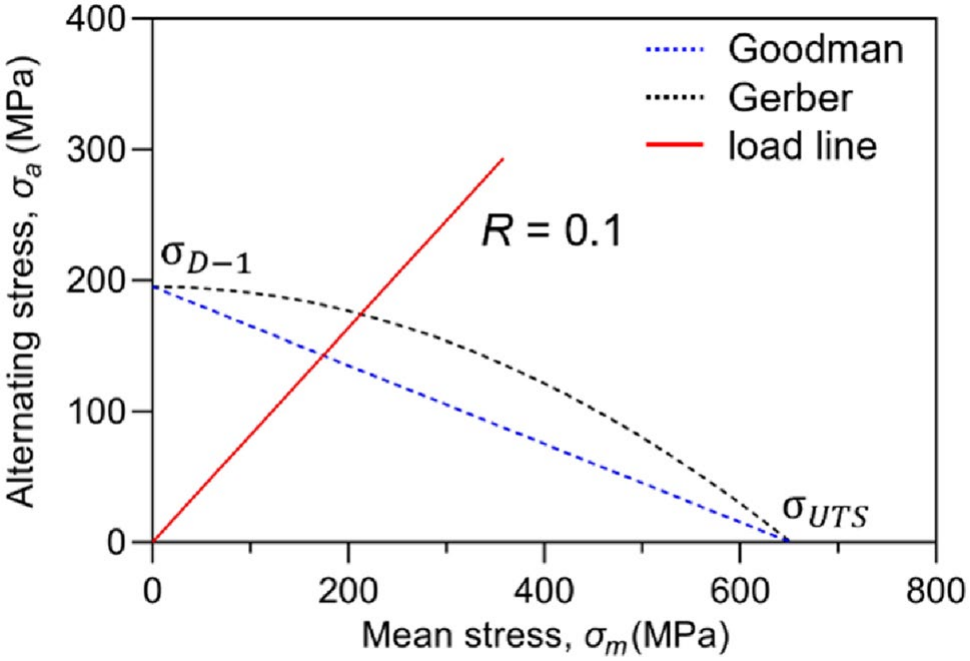
Haigh diagram of *Plate01* in pure titanium. Intersections between the load line and the applied criterion provide alternating and mean stress values for the prediction of the fatigue life

The traditional Goodman relationship and the Gerber parabola were represented in the Haigh diagram of Fig. 3 for pure titanium of *Plate01*.

The Goodman equation was used to determine ${\upsigma }_{m}$ at *R-ratio* = 0.1 as

7 $${\upsigma }_{m}=\frac{{\upsigma }_{D-1}}{{\mathrm{R}}_{a}+ {\upsigma }_{D-1}/{\upsigma }_{\mathrm{UTS}}}$$

while exploiting Gerber parabola ${\upsigma }_{m}$ was calculated as

8 $${\upsigma }_{m}=\frac{-{\mathrm{ R}}_{a}+\sqrt{{{\mathrm{R}}_{a}}^{2}+4{{(\upsigma }_{D-1})}^{2}/{{(\upsigma }_{\mathrm{UTS}})}^{2}}}{2({\upsigma }_{D-1})/{{(\upsigma }_{UTS})}^{2}}$$

where *R*_*a*_* = σ*_*a*_*/σ*_*m*_ = 0.82 at* R-ratio* = 0.1.

Afterwards, the fatigue limit under testing conditions ${(\upsigma }_{D}$) was established as

9 $$\upsigma_{D} = \text{R}_{a} \,\upsigma_{m}$$

The maximum bending moment (*M*) was calculated from the maximum stress of the plate using the following relationship (Eq. 10):

10 $$M=\frac{{\sigma }_{\mathrm{max}} {I}_{xx}}{\left(t/2\right)}$$

where $${\sigma }_{\mathrm{max}}$$ = $${\sigma }_{D}+ {\sigma }_{m}$$.

The maximum bending moment was determined for each bone plate using both Goodman and Gerber failure criteria. These values were used to define the range of the runout moment prediction.

### Verification of Predicted Bending Moments

The experimental loads obtained from fatigue testing were used to verify analytical calculations: in order to consider loads above the runout condition, stress-life (S–N) diagrams were estimated for each plate. In the case of S–N diagrams with a constant *R-ratio*, the most accurate estimation can be obtained by connecting the fatigue limit (*G*) with the low-cycle fatigue point (*F*) with a straight line. *G* has coordinates (*N*_*G*_; ${\upsigma }_{D}$), where *N*_*G*_ is the number of cycles to which the fatigue limit refers. In this case, *N*_*G*_ was equal to one million, as required for the experimental runout condition. The F point was established by assuming that the endurance limit at 10^3^ cycles was equivalent to 90% of the static failure value [20]:

11 $${\upsigma }_{F}= 0.9{\upsigma }_{\mathrm{UTS}}\frac{1-R}{2}$$

In this stage, Basquin’s equation [6] was applied to calculate the alternating stress as a function of the experimental number of cycles.

12 $${\upsigma }_{a}=A{N}^{b}$$

Constants *A* and *b* can be obtained by imposing the passage between *F* and *G* points.

**Fig. 4:**
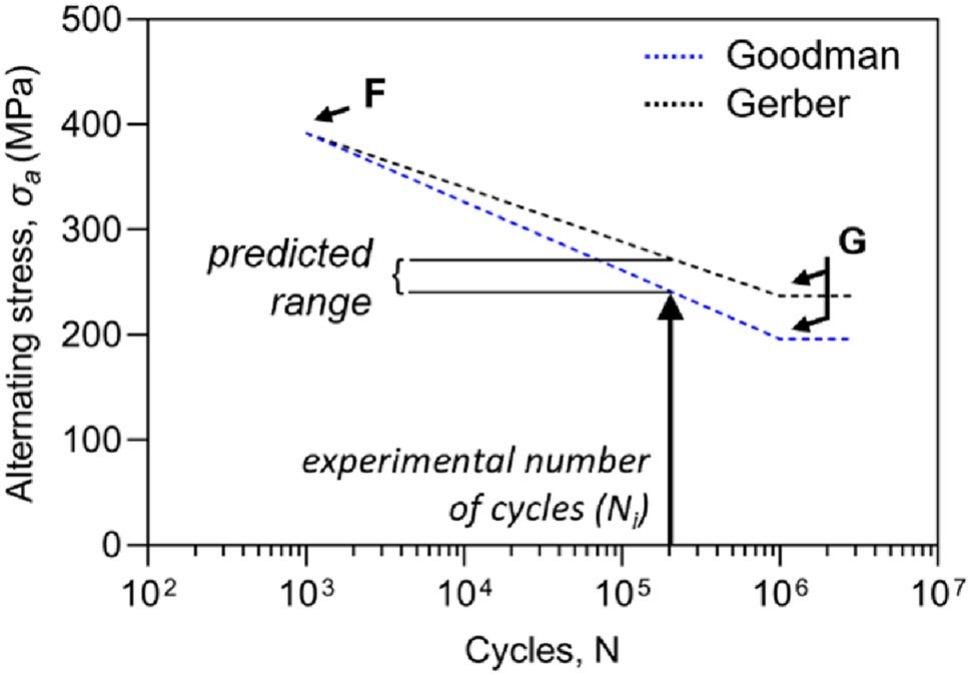
Semi-log S–N diagram of *Plate10*. *F* represents the low-cycle fatigue limit (10^3^), while *G* is the fatigue limit at 10^6^ cycles. S–N diagrams were used to verify the overall goodness of the fatigue predictions by introducing the number of cycles obtained from all the experimental tests (*N*_*i*_)

Exploiting the same F point, two curves were plotted using the fatigue limits estimated in "Analytical Fatigue Limit Determination" section with Goodman and Gerber criteria. Figure 4 displays the S–N semi-logarithmic diagrams achieved for *Plate10*.

In order to assess the accuracy of the predicted moments, S–N diagrams were applied to determine the alternating stress from an experimental number of cycles. After the calculation of the maximum stress (${\sigma }_{\mathrm{max}}$), the resulting moments were then computed through Eq. 10, and categorized into three intervals: (a) bending moments in the plastic range, (b) bending moments in the elastic range, and (c) bending moments in the runout condition. The analysis was conducted by collecting the corresponding values from all the experimental tests, including those with loads exceeding the runout, which resulted in fractured plates.

Lastly, predictions realized with the Goodman and Gerber criteria were evaluated separately for each of the three intervals through the linear least squares (LLS) calculation from the 45° line defined by experimental results (*R*^*2*^ = 1).

## Results

### Maximum Bending Moments Comparison

Table 2 displays the cross-sections of the plates and their respective properties used for the fatigue calculation. From this table, it is possible to observe how the bone plates differ in size and shape.

**Table 2:**
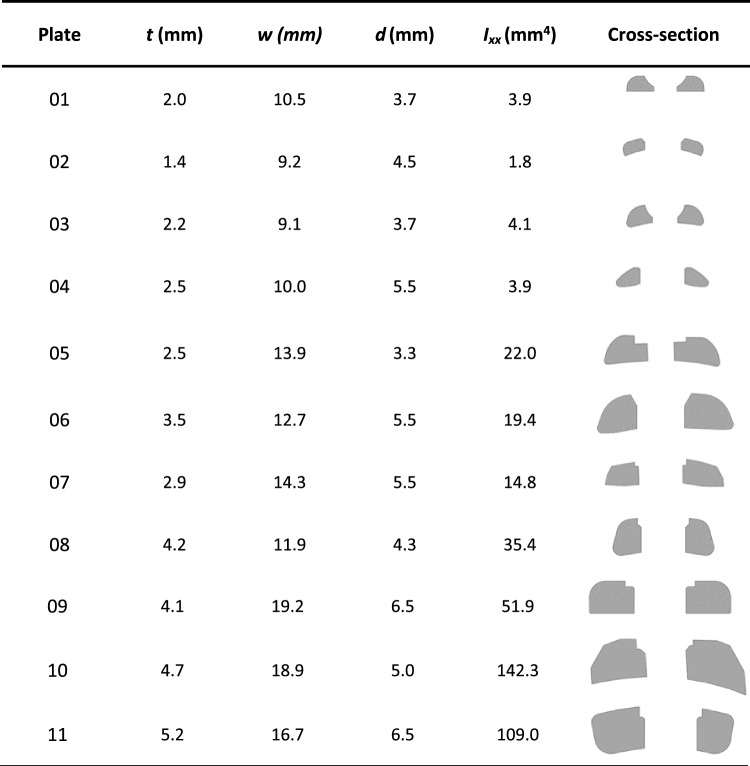
Geometric characteristics extracted from the CAD files of the bone plates involved in the study. Thickness (*t*), width (*w*), hole diameter (*d*), moment of inertia (*I*_*xx*_), and cross-section are reported. The various cross-sections have been depicted in the image respecting their original scale

**Fig. 5:**
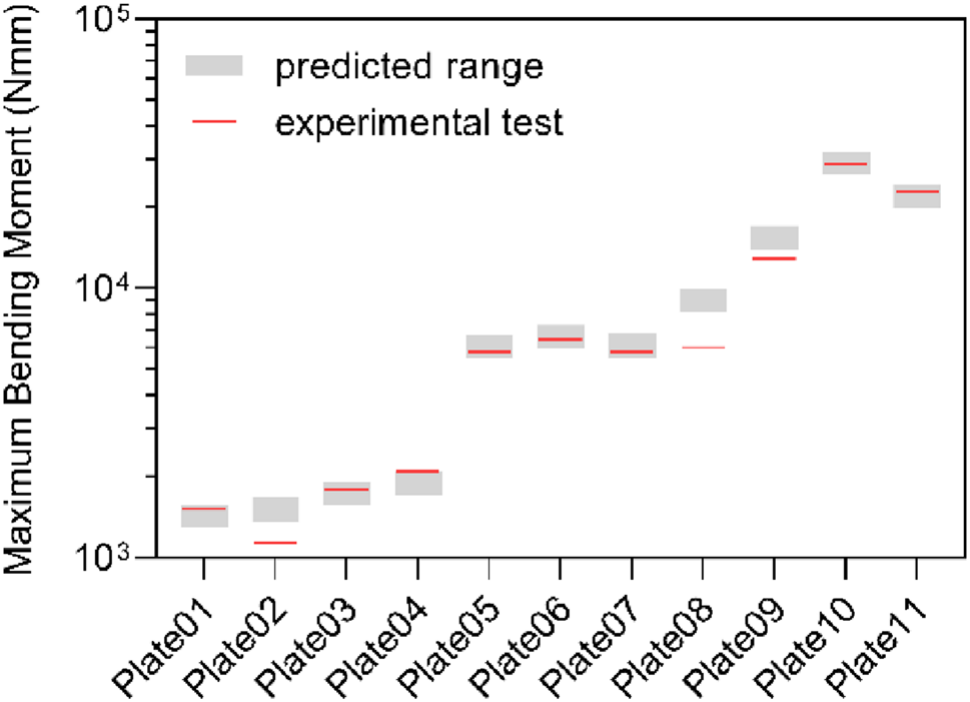
Experimental and predicted maximum bending moments obtained for each bone plate. Experimental results appear as red transversal lines, while predicted values are represented by grey boxes.

The prediction of the bending moment required to reach the runout condition for the eleven bone plates was realized and verified through comparison with experimental results obtained by applying the standard methodology described in ASTM F382. In Fig. 5, the experimental maximum bending moments (red lines) are depicted along with their predicted range of values (grey boxes).

In the runout condition, most experimental values fell within the predicted range outlined by the Goodman and Gerber criteria. On the one hand, the Goodman criterion involves a conservative life estimation: values obtained with this approach were considered as the lower limit of range prediction. Indeed, maximum bending moments calculated with the Goodman criterion lay below the experimental value in eight of the tested plates. On the other hand, the Gerber approach was useful to estimate the upper limit of the runout load: all tested bone plates showed a maximum bending moment below the limit estimated with the Gerber criterion.

However, three cases deviate from the expected fatigue values: the experimental maximum bending moments exhibited by *Plate02*, *Plate08*, and *Plate09* were below the predicted value from the Goodman criterion. Among these three plates, *Plate08* showed a maximum experimental bending moment significantly lower than predicted. Indeed, the experimental results of this bone plate were rather ambiguous: fifteen fatigue tests were performed and a load equal to approximately 30% of the static proof load was identified to reach the runout condition, suggesting a potential mismatch between the raw material certification and the material actually used to manufacture the batch of tested plates.

### Verification of Predicted Bending Moments

**Fig. 6:**
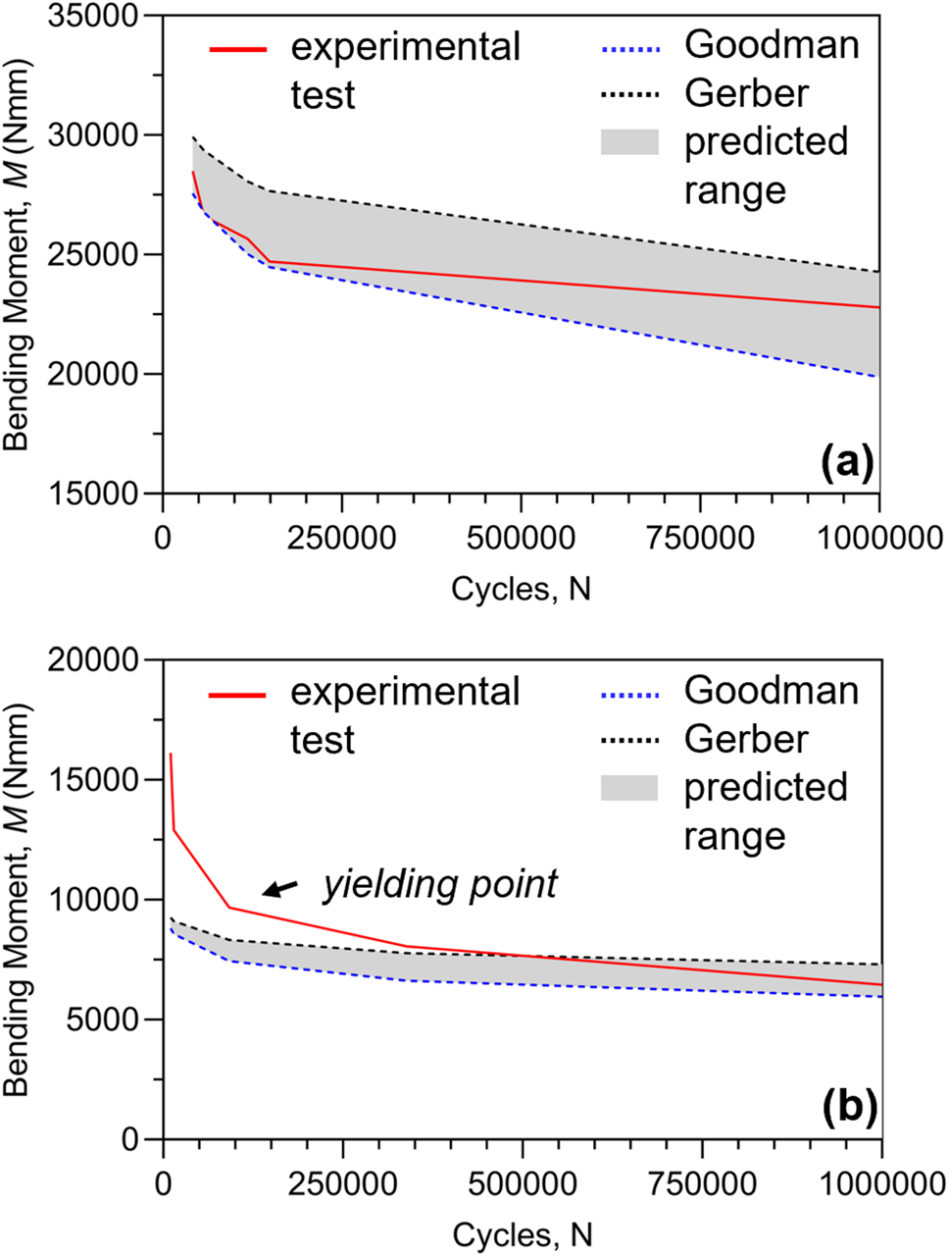
Experimental and predicted bending moments obtained for *Plate10* (**a**) and *Plate06* (**b**). Grey areas represent the predicted range of values for a generic number of cycles

S–N diagrams estimation allowed the application of all experimental data for the verification of the analytical prediction. Each experimental number of cycles was exploited to verify the analytical prediction with the experimental bending moment. By means of experimental tests, it was feasible to verify the entire trend of the prediction depending on the applied criterion (M–N diagrams). Figure 6 shows the experimental results of *Plate10* and *Plate06* compared to the analytical predictions. It is possible to observe that when all experimental loads did not generate stresses that exceed the yield strength, experimental bending moments completely fell within the predicted range (Fig. 6a). Conversely, the predicted values proved to be imprecise when the material's yield point was surpassed (Fig. 6b).

**Fig. 7:**
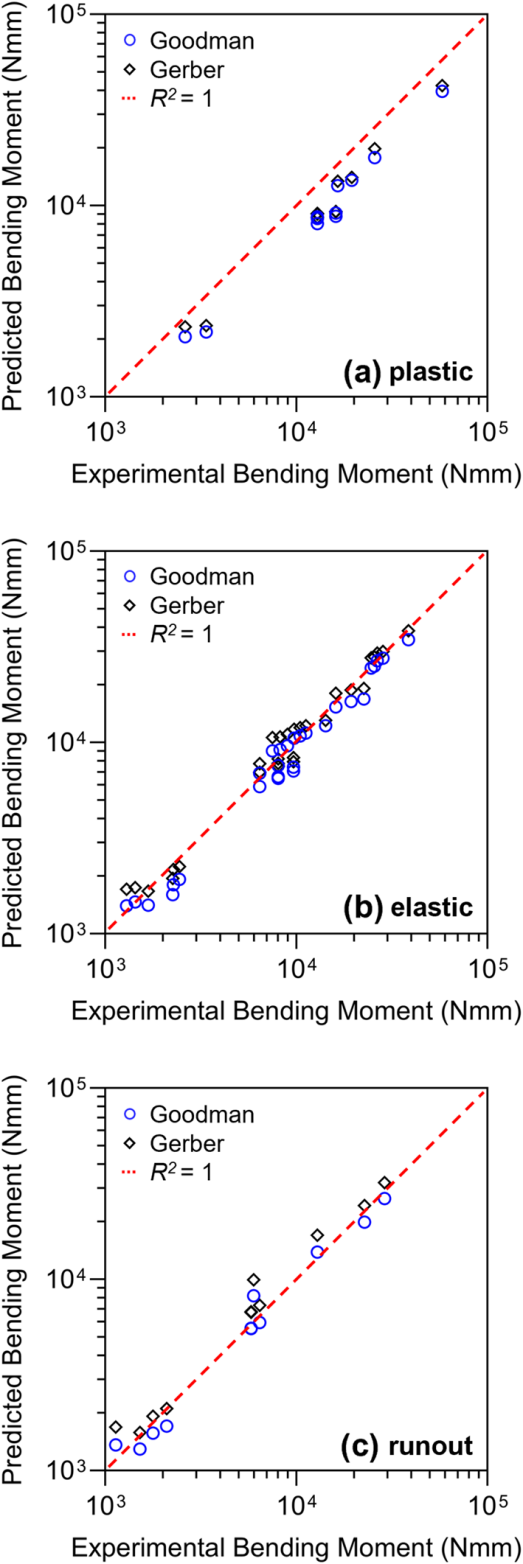
Evaluation of predicted bending moment trough linear least squares (LLS) calculated from the 45° line (red dashed) defined by the experimental results (*R*^2^ = 1). Analytical results were collected in three intervals: **a** plastic range, **b** elastic range, and **c** runout condition

LLS results according to the considered range were reported in Fig. 7. The predicted values were compared with experimental results to compute coefficients of determination (*R*^*2*^). The predictive capability drastically decreased when the yield strength of the material was exceeded (Fig. 7a). In the elastic range of the material the performance improved significantly, and the Gerber criterion better approximated the overall behaviour of the analysed plates (Fig. 7b). In the runout condition, on the other hand, similar coefficients were obtained, as the experimental value falls within the middle of the predicted range (Fig. 7c). The coefficients of determination in the three intervals are summarized in Table 3.

**Table 3:** Coefficients of determination in plastic, elastic and runout conditions for Goodman and Gerber criteria

	*R*^2^		
	Plastic	Elastic	Runout
Goodman	0.409	0.960	0.969
Gerber	0.618	0.972	0.955

## Discussion

In this study, an analytical procedure was presented to support standard mechanical testing starting from the ultimate tensile strength specified in the raw material certification and the bone plate geometry. The analytical predictions of eleven non-certified bone plates were then compared with experimental results.

Stress-life approaches have been developed and widely used in the aerospace field [6], while applications in the biomedical field are not as widespread. These approaches were recently employed in mandibular plates [17, 28] and dental implants [29]. However, in these studies, the input values were obtained from Finite Element (FE) analysis, a technique widely validated in the biomedical field but expensive in terms of time and costs (for example the costs associated with licenses for commercial use, which not all companies have, and computational time). For this reason, our goal was to implement a fast and low-cost algorithm that can be used as a support for experimentation.

Kenedi and Vignoli [11] conducted in 2017 an analytical study on the behaviour of osteosynthesis plates, focusing on the static condition. Nevertheless, to the best of the authors' knowledge, no study has been conducted to provide support for the experimental testing of bone plates required for regulatory purposes.

Due to the phenomenology of fatigue, it is difficult to estimate a unique fatigue limit with a simple procedure. Indeed, even if the ASTM standard claims the identification of the runout load by gradually decreasing the static proof load, analytical models can be adopted to reduce the range of loads to be investigated in standard fatigue testing. Employing a frequency of 5 Hz to conduct fatigue tests, the three tests at one million cycles require approximately 167 hours to be completed. Each additional test conducted to identify the runout load involves numerous hours spent and specimens utilized. The fatigue tests for the eleven tested plates lasted on average about 205 h. Through a comparison of the experimental value of the maximum bending moment with the upper limit of the predicted range (Gerber criterion), it was possible to estimate the potential time savings achievable by directly employing the analytical procedure to complement the experimental tests. By excluding all loads above the upper limit, approximately 10% of the hours spent could have been saved. The worst-case scenario in terms of unproductive time was observed for *Plate04*, as three tests with a total duration of nearly 50 hours (23% of the total test duration) could have been excluded. Moreover, these tests correspond to additional specimens that the manufacturer must provide for testing purposes: applying the analytical procedure would have saved a total of twenty-seven specimens. The worst-case scenario was encountered during the tests conducted for *Plate06*, where four specimens could have been saved if the analytical approach had been adopted directly. In addition, it was found that in eight cases, the initial fatigue test was performed with loads that generated stresses over the yield strength. These tests would have been excluded *a priori*.

Furthermore, it is important to note that the experimental runout condition does not correspond to a plate fracture at one million cycles, but to the first test where, by gradually reducing the load, one million cycles were reached without breaking. Thus, as the runout condition does not correspond to a specimen failure, there remains uncertainty regarding whether the specimen would have failed after one million cycles plus one. In fact, the standard procedure entails gradually reducing the load in discrete steps until reaching runout (i.e., an intact plate at a million cycles). As a result, the identified load would undoubtedly have caused plate failure in a higher number of cycles (to a varying degree) than the runout. This would imply the need to increase the load again in smaller amplitude steps until the one corresponding to a one million cycles rupture is identified. Therefore, in all experimental runouts the load is always underestimated, more or less severely. In this context, the analytical procedure could provide a preventive indication of the runout load in order to avoid excessive underestimation. Results reported for *Plate08* were useful to analyse the possible implication of fatigue life estimation before starting a test campaign. Although more than 250 hours have been spent testing this bone plate, the maximum bending moment was most likely underestimated because the decrement from the previous test was probably too large. Compared to the other tibia plates, *Plate08* was expected to have higher performance due to the larger moment of inertia of its cross-section. If the test had been conducted using the analytical procedure, it would have been possible to use a smaller load decrement since the lower limit of the predicted range (Goodman criterion) would have served as a reference.

In addition to the indication on the runout, the analytical prediction allows for a comparison of the entire test results, ensuring that the properties of the plate align with the expectations from the beginning. Compared to the values related to the fatigue limit, the validity of estimated diagrams for limited durations is more uncertain [20]. Although the fatigue strength in low-cycle regions is around 90% of the ultimate strength, the real stress level is not so high. The rationale behind this is that the fatigue strength values corresponding to the experimental data points were derived based on an elastic formulation (Eq. 10). Load levels significant enough to cause failures in a thousand cycles usually induce substantial plastic deformation. Consequently, when the yield point is exceeded, the real stress levels are lower than the calculated values [10].

Some limitations are present in this study concerning (1) the calculation of the fatigue-notch factor (${K}_{\mathrm{f}}$) for titanium plates. Indeed, for these plates ${K}_{\mathrm{f}}$ was considered equal to ${K}_{t}$ from a conservative perspective. This approximation has led to an overestimation of the stress present in the hole and, consequently, to a lower estimation of the expected number of cycles; and (2) neglecting the curvatures present in the cross-section for the calculation of ${K}_{t}$, which may have caused an underestimation of stresses. To overcome these limitations, it would have been necessary to (1) conduct a fatigue experimental campaign on standardized samples for each type of titanium used, and (2) implement a virtual FEM-based design of experiment to estimate a corrective coefficient from rectangular plates of various curvatures with respect to Peterson's traditional stress concentration factors [18].

Moreover, no indication is provided by the standard regarding the fatigue strength that the bone plate should guarantee in the runout condition. The determination of the minimum level of *in vivo* performance that the plate must fulfil remains an open issue, which is frequently addressed by the direct comparison with predicate devices. To tackle this, the maximum stress of the implanted bone plate could be calculated through an in silico FE twin, in order to predict the corresponding number of cycles within the here presented framework and thus define a rationale able to further reduce the regulatory process time.

In conclusion, this work reported promising results showing how the prediction of a bending moment range of values could allow the accomplishment of mechanical tests while reducing the actual testing hours and using a limited number of specimens, with a substantial impact on the costs for the manufacturer. The implemented procedure allows to realize a complete estimation of the fatigue behaviour of the fixation device starting from the ultimate tensile strength specified in the raw material certification and bone plate geometry. Moreover, the proposed analytical framework can provide a starting point for fatigue testing and could represent a guideline to avoid the underestimation of the runout load.

The procedure has already been completely automated, and a user-friendly interface will be implemented to upload the CAD file of the plate and the ultimate tensile strength of the raw material certification. This will allow the final user to obtain an immediate prediction of the performance of the plate they are designing or testing. Further works on new bone plates are planned to verify the effective reduction of test execution times through the proposed procedure.

## Figures and Tables

**Fig. 8:**
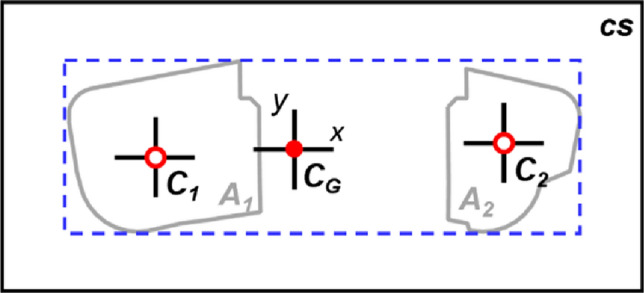
Critical section (cs) example identified with the slicing technique. The solid red circle indicates the centroid of the entire section; empty red circles represent the centroids of the polylines (in grey)

## Appendix

To determine the area (${A}_{i}$) and the centroids coordinates ${C}_{i}$(${X}_{Ci}$;${Y}_{Ci}$) of each section derived from the slicing procedure, the function *PolygonMoments* was exploited (Fig. 8). At the location of a hole, the resulting section will consist of two distinct polylines. By applying Varignon's theorem, the centroid coordinates $G$(${X}_{G}$;${Y}_{G}$) of the entire section were determined as:

$${S}_{x}={A}_{1}{Y}_{C1}+{A}_{2}{Y}_{C2} {S}_{y}={A}_{1}{X}_{C1}+{A}_{2}{X}_{C2}$$

$${X}_{G}=\frac{{S}_{y}}{{A}_{1}+ {A}_{2}}{ Y}_{G}=\frac{{S}_{x}}{{A}_{1}+ {A}_{2}}$$

where ${S}_{x}$ and ${S}_{y}$ were the section static moments.

The Huygens-Steiner theorem can be employed to ultimately determine the centroidal moment of inertia of the entire section relative to the bending axis (${I}_{xx}$):

$$\begin{gathered} I_{xCG1} = I_{x1} + A_{1} \left( {Y_{CG} - Y_{C1} } \right)^{2} \,\,\,\,\,\,\,\,I_{xCG2} = I_{x2} + A_{2} \left( {Y_{CG} - Y_{C2} } \right)^{2} \hfill \\ I_{xx} = I_{xCG1} + I_{xCG2} \hfill \\ \end{gathered}$$

## List of Symbols

*σ*_max_: Maximum stress
*σ*_min_: Minimum stress
*σ*_*a*_: Stress amplitude
*σ*_m_: Mean stress
*R*-*ratio*: *σ*_Min_/*σ*_max_
*R*_a_-*ratio*: *σ*_*a*_/*σ*_*m*_
*σ**_*D*__−__1_: Specimen stress amplitude for *σ*_*m*_ = 0 (*R*-*ratio* = − 1)
*σ*_*D*__−__1_: Component stress amplitude for *σ*_*m*_ = 0 (*R*-*ratio* = − 1)
*σ*_UTS_: Ultimate tensile strength
